# Motivational interviewing and urine cotinine feedback to stop passive smoke exposure in children predisposed to asthma: a randomised controlled trial

**DOI:** 10.1038/s41598-017-15158-2

**Published:** 2017-11-13

**Authors:** Sasha G. Hutchinson, Gerard van Breukelen, Constant P. van Schayck, Brigitte Essers, S. Katharine Hammond, Jean W. M. Muris, Frans J. M. Feron, Edward Dompeling

**Affiliations:** 1 Department of Paediatric Pulmonology, Maastricht University Medical Center (MUMC+)/CAPHRI School for Public Health and Primary Care, P.O. Box 616, 6200 MD Maastricht, The Netherlands; 2 Department of Methodology and Statistics, MUMC+/CAPHRI, P.O. Box 616, 6200 MD Maastricht, The Netherlands; 3 Department of Family Medicine, MUMC+/CAPHRI, P.O. Box 616, 6200 MD Maastricht, The Netherlands; 4 Department of Clinical Epidemiology and Medical Technology Assessment, MUMC+, P.O. Box 616, 6200 MD Maastricht, The Netherlands; 5 School of Public Health, University of California, Mail/140 Warren, Berkeley, CA 94720–7360 USA; 6 Department of Social Medicine, MUMC+/CAPHRI, P.O. Box 616, 6200 MD Maastricht, The Netherlands

## Abstract

We tested the effectiveness of a program consisting of motivational interviewing (MI) and feedback of urine cotinine to stop passive smoking (PS) in children at risk for asthma. Fifty-eight families with children 0–13 years with a high risk of asthma and PS exposure were randomised in a one-year follow-up study. The intervention group received the intervention program during 6 sessions (1/month) and the control group received measurements (questionnaires, urine cotinine, and lung function) only. The primary outcome measure was the percentage of families stopping PS (parental report verified and unverified with the child’s urine cotinine concentration <10 μg/l) in children during the intervention program. The analyses were performed with Mixed Logistic Regression. After 6 months, a significant group difference was observed for the unverified parental report of stopping PS in children: 27% of parents in the intervention group versus 7% in the control group. For the verified parental report, the difference was similar (23% versus 7%) but was not statistically significant. Despite a limited sample size, the results suggest that the intervention program is probably an effective strategy to stop PS in children. A program longer than 6 months might be necessary for a longer lasting intervention effect.

## Introduction

Asthma is the foremost chronic disease in children and is associated with increased morbidity and decreased quality of life^1^. Children with a first degree relative (biological parent or sibling) with asthma have a higher risk of developing asthma themselves than children without a genetic predisposition^2^. Environmental factors like passive smoking (PS)^3^ are also associated with the incidence of asthma in children due to interaction between genes and environment^4,5^. PS in children has been significantly associated with increased incidence of asthma in children^6^. Therefore, the prevention of PS in children with a high risk of asthma is of great importance.

According to the WHO, 40% of children are exposed to PS globally^7^. In the Netherlands, at least 29% of all children of families with low social economic status are exposed^8^. A recent meta-analysis of interventions to protect children against PS concludes that effective intervention strategies to stop PS in children are still needed^9^. Positive effects on stopping PS exposure in children have been described for behavioural counselling methods, such as motivational interviewing (MI)^10,11^. Emmons *et al*. evaluated the efficacy of MI for reducing household PS exposure in young children and found significantly lower nicotine concentration levels in the MI group compared to the control group after 6 months of study^12^. Nurse-led sessions employing behaviour changing strategies with repeated feedback on the child’s urine cotinine levels resulted in reduced concentrations of urine cotinine in children, increased proportion of parents quitting smoking and a significant decrease in asthma related visits to health care providers^13^. A Dutch study showed that reduction of PS exposure as part of a multiple intervention strategy in children with high risk of asthma was not effective, indicating that intervention strategies towards stopping PS in children should be focused and not part of larger multiple intervention programs^14^. Furthermore, previous studies showed that brief interventions to stop PS exposure in children may not be effective^15,16^. Therefore, we hypothesised that an effective intervention to stop PS in children with a high risk of asthma may be possible by incorporating elements of previous trials into one intervention program^12,13,16–20^. Such intervention program should consist of a tailored MI program with repeated contacts at home, focusing on awareness, education, perceived barriers, perspectives and needs of parents, in combination with feedback about urine cotinine levels of the children. As brief interventions were not effective, we choose 6 MI sessions during 6 months. More details about the PREPASE (PREvention of PAssive Smoking Exposure) intervention have been published before^21^. The aim of the PREPASE study was to evaluate the effectiveness of such an intervention.

## Methods

The PREPASE study is a 1 year randomised controlled trial conducted in Limburg, the Netherlands, at the Maastricht University Medical Centre (MUMC+). The study was approved by the medical ethics committee MUMC+ and we confirm that all methods were performed in accordance with the relevant guidelines and regulations. The study protocol has been published previously^21^. The PREPASE study was registered at the Dutch Trial Register (www.trialregister.nl) on November 29, 2010. Registration number: NTR2632.

### Participants

**Figure 1:**
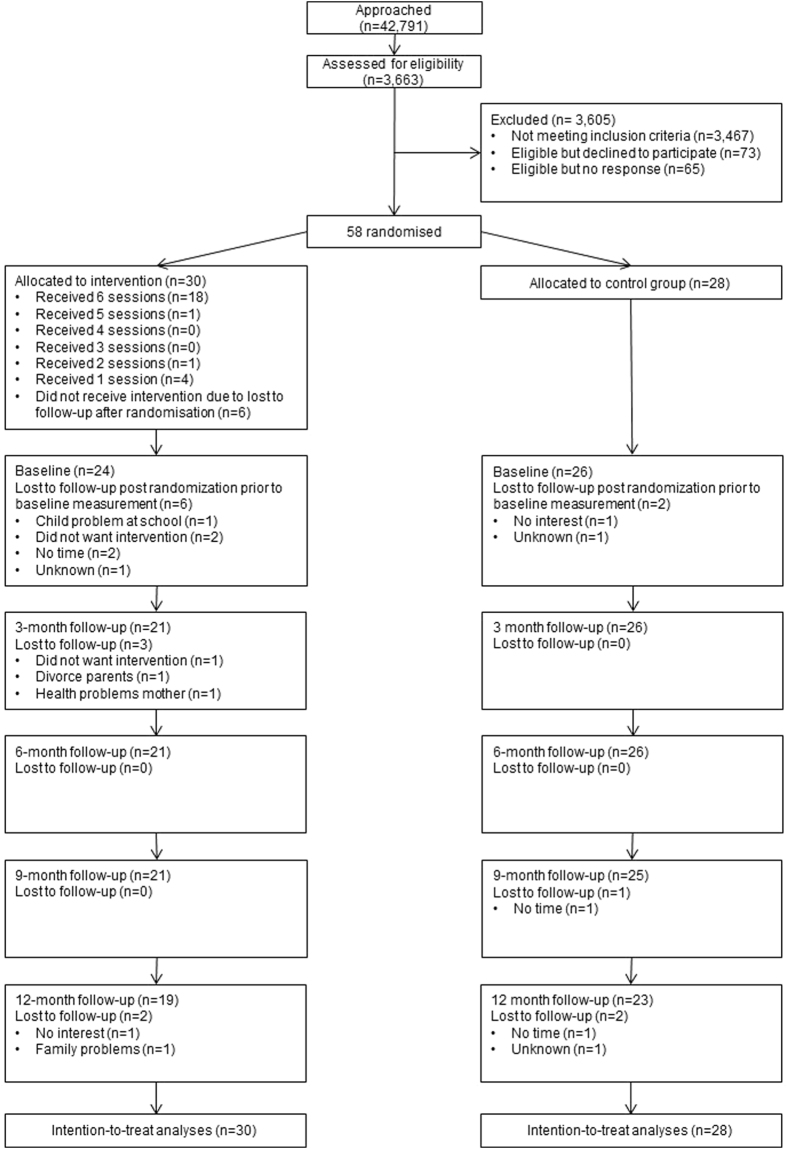
Flow Chart. Flow chart randomised controlled trial.

Families were eligible to participate when their youngest child was aged ≤13 years, had a high risk of asthma (due to a positive family history of asthma in the first degree (father/mother/sibling)), and was exposed to PS at home. Families were not eligible to participate when the child actively smoked, had a chronic lung disease, when the child/parent(s) had mental retardation, or, when the family had professional help for smoking cessation. Participants for the study were recruited via: 1) a questionnaire that was distributed to their home addresses by the family physicians or the research team, 2) an electronic survey distributed via primary schools of children, 3) other studies^22,23^, 4) actively via physicians working in child-care settings, 5) the youth health care department of the Regional Public Health Service, and 6) advertisements in a local newspaper, child-care facilities and day-care centres. More than 42,791 families were approached. All families received a (electronic) questionnaire on respiratory complaints of their children and smoking behaviour of the parents. The questionnaires were filled in by both parents and were used to assess if the families were eligible to participate in the study. In case they were eligible and provided permission to be contacted, they were then contacted by phone to ask if they were willing to participate in the RCT. Of all the families approached, only 3663 participated with the questionnaires. Thereof, only 196 families met our inclusion criteria (Fig. 1); 73 families declined to participate whereas 65 gave no response, leaving 58 families to be randomised.

### Randomisation

After written informed consent was received, eligible families were randomised by a randomisation system that was built in the study’s logistic system which automatically randomised the participants with an allocation ratio of 1^21^. The families were pre-stratified according to the age of the children (<6 years and ≥6 years) to prevent imbalances between the intervention and the control group that may be caused due to variables related to the age of the children^21^. Prior to randomisation, limited information was given concerning the intervention^21^. All families were informed that we were testing a program to help parents prevent PS in their children and to study the association between PS and respiratory complaints in children. After randomization, the control group remained unaware of the intervention program and received measurements only. Additionally, the control group did not receive feedback about the children’s urine cotinine level. The research group and follow-up assessors were aware of the group allocation.

### Intervention

The MI intervention consisted of 6 home-based sessions with the parents once per month for about 60 minutes. An extensive description of the intervention program and the study protocol has been published previously^21^. The intervention program was based on the principles of the reasoned action model and consisted of effective elements of previous interventions; being motivational interviewing with repeated contacts aimed at motivating parents to stop PS in children^12,24^. The sessions were focused on increasing parental motivation and confidence to stop PS in their children. The counsellors were encouraged to provide empathy to the parents and not to criticize them. The sessions were tailored to the parents’ personal goals to stop PS in their children. Parents received feedback on the children’s urine cotinine concentrations during the second, fourth and last sessions to increase parental awareness of PS in children. When applicable, both parents were encouraged to participate in the intervention program, regardless if they both smoked or not.

### Training

Both counsellors were already trained in MI. For this study, they received an extra training by an expert from STIVORO (Dutch expert centre on tobacco control) about smoke addiction, health effects of PS in children, measures to prevent PS in children and advanced training on MI. Further coaching was given by an experienced MI trainer from the MUMC+. A counselling protocol was developed to serve as a guideline during the sessions^21^.

### Primary outcomes

The primary outcome parameter was the percentage of families stopping PS exposure in their children, which was compared between the intervention and the control group at different time points. PS in children was measured via parental reports and checked with the urine cotinine concentrations of the children. Two primary outcomes measures were used: first, parental report of no PS in their children at home as verified by the child’s urine cotinine concentration being <10 μg/l. Second, parental report of no PS without urine cotinine verification. The reason for this was two-fold: first, urine cotinine results were used as feedback to the parents as part of the intervention in the intervention group, implying overlap between outcome and treatment; second, if urine cotinine was missing, PS was assumed to be present for the first primary outcome, which may be a too strict/pessimistic criterion, hence the second primary outcome. The second outcome therefore also served to check the robustness of the results against this assumption.

The time-point 6 months was used to measure the primary outcome directly after the 6 months intervention. Furthermore, the other time-points were used to evaluate the difference between the intervention and the control group at each time-point.

### Urine cotinine measurement

On the day of measurements, all parents were asked to collect early morning urine samples of their children. This was done at baseline, 3, 6, 9 and 12 months of study. The samples were analyzed for cotinine at Medical Laboratory Humicon B.V., Maastricht, the Netherlands, using gas chromatography–mass spectrometry technique (Thermo Scientific DSQIII, Axel Semrau GmbH & Co. KG, Sprockhövel, Germany). The laboratory was blinded to subjects’ identity and group assignment.

### Secondary outcomes

The number of cigarettes smoked per day in the presence of the child as well as actively per family was measured via questionnaires and compared between the two groups at baseline, 3, 6, 9 and 12 months. For the analysis cigarettes and roll ups were considered equal, a cigar was treated as equivalent to four cigarettes^25^. None of the parents reported smoking the pipe.

Parental stage of change for stopping active smoking were checked and compared at baseline, 6 and 12 months of study.

Respiratory symptoms/complaints and quality of life (FSII)^26^ of the children were measured at baseline, 3, 6, 9, and 12 months of study with questionnaires. Respiratory symptoms included at least one episode of wheezing and/or respiratory tract infections in the past 3 months. ≥6 years of age. Percentages of the predicted values of the Forced Expiratory Volume in one second (FEV_1_), Forced Vital Capacity (FVC) and Maximal Expiratory Flow at 50% (MEF_50_) were used for analysis. The MicroRint (Micro Medical, Rochester Ltd, UK) was used in children <years of age (n=6). The median MicroRint values were used for analysis.

The intervention was evaluated at the end of the study by means of a questionnaire assessing parents’ opinion regarding the content of the intervention program, the counselling style and the counsellors.

### Statistical analyses

The statistical analyses were performed with the Statistical Package for Social Sciences (SPSS) version 20. The sample size calculation has been published before^21^. The descriptive statistics of the baseline variables were tabulated per research group. We checked for differences on the baseline characteristics between the intervention and control group, using the Chi-square contingency test for categorical variables, respectively the independent samples Student t-test for continuous variables with a near normal distribution and no outliers, and the Mann-Whitney U-test else. Due to the randomised assignment, no significant baseline differences were expected apart from those due to multiple testing, and so these tests served to check the randomisation procedure as well as selective drop-out before baseline measurement.

The intervention effect on the primary outcomes (urine cotinine (un-) verified parental report of PS (yes (1) and no (0) in their children at home) were analysed with mixed logistic regression analysis (using Generalized Linear Mixed-effects Models (GLMM) in SPSS) of all repeated measures to allow inclusion of all participants with at least one measurement of exposure and to take the correlations between the repeated measurements into account, as the main analysis, using a two-tailed α = 0.05^27^. Missing outcome values were not imputed, because, under the so-called Missing at Random (MAR) assumption made by multiple imputation, multiple imputation is essentially equivalent to complete cases analysis in a cross-sectional setting with one outcome^28^, and equivalent to the present likelihood based inference for longitudinal studies with dropout^29^. More details of the GLMM analysis are given in the ‘Supporting information’.

## Results

### Participants’ characteristics

Fifty-eight families were randomised in the trial: 30 to the intervention and 28 to the control group. The flow of the participants is shown in Fig. 1. Immediately after randomisation, 6 families from the intervention group and 2 from the control group dropped out of the study and no variables could be recorded for these subjects apart from the child’s age, gender and exposure to PS (which was ‘yes’, as this was an inclusion criterion). However, these 8 families were included in the intention-to-treat analysis. Important baseline characteristics of the participants are presented in Table 1. No significant differences at baseline were found between the intervention and the control group. The primary caregivers and their partners in the intervention group both had a mean of 4.5 on the Fagerström test, demonstrating considerable nicotine dependence at the start of the study. Only a few parents were in the stage of preparing to quit smoking at baseline.

**Table 1:** Baseline characteristics.

	Intervention group (n = 30)^a^	Control group (n = 28)^a^
Children’s mean age in years (mean, (SD))	8.45 (2.69)	8.76 (2.42)
**Children’s gender (n, (%))**		
• Male	17 (57)	16 (57)
• Female	13 (43)	12 (42)
**Children’s respiratory complaints (n, (%)):**		
• Respiratory infection (past 12 months)	13 (43)	15 (54)
• Wheezing ever	9 (30)	11 (39)
• Recent wheeze (past 3 months)	7 (23)	8 (29)
• Dyspnea ever	5 (17)	8 (29)
• Use of inhaled drugs	2 (7)	5 (18)
**Children’s lung function:**		
• FEV_**1**_ (% predicted value pre- bronchodilator, (SE))	94.70 (2.53)	92.79 (2.15)
• FEV_**1**_ (% predicted value post- bronchodilator, (SE))	99.55 (2.57)	95.38 (1.87)
• FVC (% predicted value pre- bronchodilator, (SE))	93.25 (2.31)	90.63 (2.49)
• MEF_**50**_ (% predicted value pre- bronchodilator, (SE))	77.35 (3.62)	81.71 (4.04)
• FEV1/FVC (% pre-bronchodilator, (SE))	86.35 (1.30)	87.25 (1.48)
**Primary caregiver (n, (%)):**		
• Mother	21 (70)	21 (75)
• Father	3 (10)	5(18)
**Mean age primary responder in years (mean**, **(SD))**	37.17 (6.11)	42.15 (6.62)
**Single parent families (n, (%))**	3 (10)	3 (11)
**Smoke behavior parent(s) (n, (%)):**		
• Smoking mothers	9 (30)	9 (32)
• Smoking fathers	7 (23)	7 (25)
• Smoking both parents	8 (27)	10 (30)
**Parental cigarettes smoked daily per family (mean, (SD))**	23.79 (16.08)	24.58 (16.14)
**Number of parental cigarettes smoked indoors**	12.17 (11.16)	12.08 (13.83)
**Urinary cotinine child in μg/l (mean, (SD))**	9.03 (11.71)	13.12 (20.46)
**Nicotine dependence, FTND (mean, (SD)):**		
• Primary caregiver	4.45 (2.31)	3.74 (2.62)
• Partners	4.45 (2.70)	3.82 (2.27)
**Stages of change (n, (%))**		
**•** ***Primary caregiver***		
No intention	10 (42)	7 (27)
Precontemplation	4 (17)	3 (12)
Contemplation	4 (17)	6 (23)
Preparation	2 (8)	3 (12)
Not applicable (non-smoker)	4 (17)	7 (27)
**•** ***Partners***		
No intention	7 (29)	11 (42)
Pre-	1 (4)	0 (0)
contemplation	3 (13)	4 (15)
Contemplation	1 (4)	2 (8)
Preparation	12 (50)	9 (35)
**Highest parental education (n, (%)):**		
• Low	4 (13)	4 (14)
• Middle	15 (50)	13 (46)
• High	5 (16)	9 (32)

^a^Except for the child’s age and gender, baseline characteristics were not available for n = 6 families in the intervention group and n = 2 families in the control group due to dropout (lost- of-follow up) before baseline measurement.

Test of the baseline group difference: p > 0.05 for all variables, indicating absence of severe bias due to dropout.

### Completeness of parental participation in counselling sessions

Sixty percent (n=18) of the families in the intervention group participated in all counselling sessions; 3% (n=1) participated in 5 sessions and missed 1 session due to parental lack of time; 3% (n=1) stopped counselling after 2 sessions due to stopping active smoking; 13% (n=4) participated in 1 counselling session, for 1 family counselling was no longer necessary because the mother separated from her partner who was the smoker, and the remaining 3 families were lost to follow-up; 20% (n=6) families did not participate in any counselling session because they dropped out immediately after randomisation (Fig. 1).

### Intervention effect

The course of verified and unverified parental report of PS in children at home is presented in Table 2. Both primary outcome definitions showed a difference in favour of the intervention group. After 3 months, there were no significant differences between the two groups (p>0.10) for either primary outcome definitions. After 6 months, stopping PS in children was reported in 23% (cotinine verified) or 27% (unverified) of the families in the intervention group compared to 7% (for both cotinine outcomes) of the families in the control group. The GLMM showed a borderline significant difference between the two groups for the cotinine verified parental report of PS (p<0.10), and a significant difference between the two groups for the unverified parental report of PS (p<0.05). After 9 months, the group difference was significant for the unverified parental report of PS according to the GLMM (the cotinine verified report gave an extreme standard error due to absence of any success/exposure stop in the control group, see Table 2). After 12 months of study, none of the test results was significant (all p>0.10 two tailed). The results of the GLMMs are provided in Table 3. For more results, see the ‘Supporting information’.

**Table 2:** Passive smoking verified and not-verified by urine cotinine in the intervention and control group in the course of time.

Time	Passive					
	Intervention group (n (%))			Control group (n (%))		
	No	Yes	Missing*	No	Yes	Missing*
***Verified by urine cotinine***						
0 months	0 (0)	30 (100)	0 (0)	0 (0)	28 (100)	0 (0)
3 months	3 (10)	17 (57)	10 (33)	0 (0)	21 (75)	7 (25)
6 months	7 (23)	11 (37)	12 (40)	2 (7)	19 (68)	7 (25)
9 months	4 (13)	13 (43)	13 (43)	0 (0)	23 (82)	5 (19)
12 months	5 (17)	13 (43)	12 (40)	2 (7)	19 (68)	7 (25)
***Not-verified by urine cotinine***						
0 months	0 (0)	30 (100)	0 (0)	0 (0)	28 (100)	0 (0)
3 months	3 (10)	17 (57)	10 (33)	0 (0)	21 (75)	7 (25)
6 months	8 (27)	10 (33)	12 (40)	2 (7)	19 (68)	7 (25)
9 months	7 (23)	10 (33)	13 (43)	2 (7)	21 (75)	5 (19)
12 months	6 (20)	12 (40)	12 (40)	4 (14)	17 (61)	7 (25)

*‘Missing’ include lost to follow-up as seen in Fig. 1 as well as families who were not lost to follow-up but were not able to participate with one/more of the measurements.

**Table 3:** Model estimates^a^ for group difference between intervention and control group (reference) for urine cotinine verified and not-verified parental report of passive smoking in children.

Variable	Estimate (β)	Standard Error	p-value (two- tailed)	Odds Ratio intervention/control resp. control/intervention
***Passive smoking verified (unadjusted*** ^**b**^ **)**				
Time = 3 months	−3.83	3.01*	0.21*	0.02 resp. 29.37*
Time = 6 months	−1.72	0.92	0.06	0.18 resp. 5.60
Time = 9 months	−7.78	14.40*	0.59*	0.00 resp. 2,392*
Time = 12 months	−1.30	0.93	0.17	0.27 resp. 3.67
***Passive smoking not-verified (unadjusted*** ^**b**^ **)**				
Time = 3 months	−5.06	4.65*	0.28*	0.01 resp. 156.80*
Time = 6 months	−1.88	0.87	0.03	0.15 resp. 6.53
Time = 9 months	−1.96	0.93	0.04	0.14 resp. 7.11
Time = 12 months	−0.57	0.74	0.44	0.56 resp. 1.77

Odds ratio of exposure for treated versus control respectively for control versus treated. ^a^GLMM, dependent variable passive smoking (yes (1) or no (0)).

^b^Unadjusted: analysis without gender and age as covariates.

*Large Standard Error, p-value and Odds Ratio due to zero non-exposed in the control group (see Table 2).

### Number of cigarettes smoked in the presence of the child

**Figure 2:**
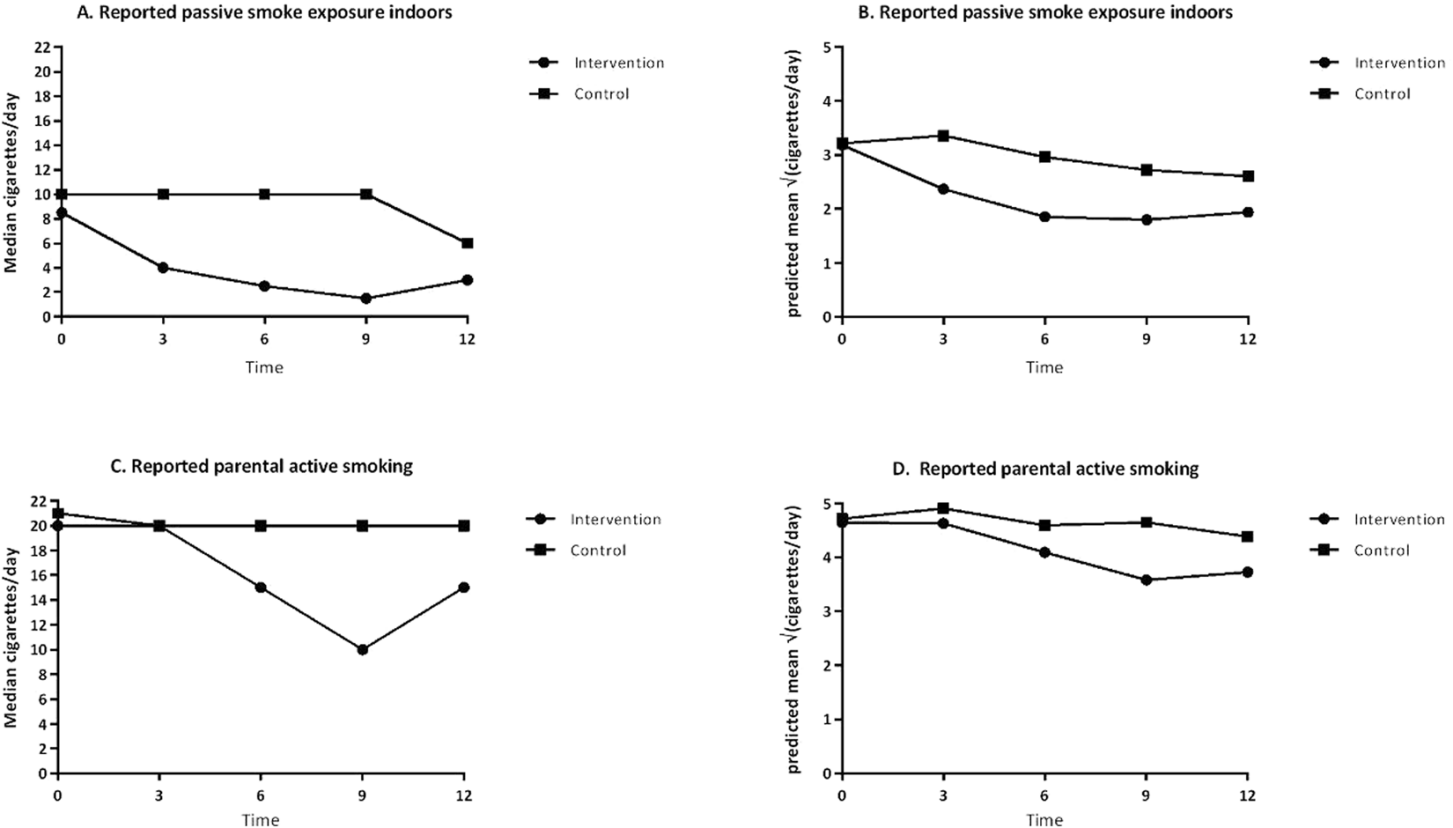
Amount of cigarettes smoked in the presence of the child indoors and amount of cigarettes smoked by parents (actively). (**A**) Median amount of cigarettes smoked in the presence of the child indoors. (**B**) Predicted mean square root of the amount of the cigarettes smoked in the presence of the child indoors, based upon the Generalized Linear Mixed-effects Models (GLMM) model analysis of all available data after square root transform and without imputing missing values. (**C**) Median amount of cigarettes smoked by parents (active smoking). (**D**) Predicted mean square root of the amount of the cigarettes smoked by parents based upon the GLMM analysis of all available data after square root transform and without imputing missing values.

The number of cigarettes smoked in the presence of the child decreased over time in both groups. In view of strong skewness of this outcome, we plotted the median rather than the mean of this outcome against time per group (Fig. 2), showing that the number of cigarettes smoked in the presence of the child decreased at 3, 6, and 9 months of study in the intervention group, but remained constant in the control group. The difference in the medians from baseline to 6 months of study was −5.5 cigarettes in the intervention group and 0 cigarettes in the control group (Fig. 2 panel A). Mixed linear regression analysis of this outcome (square root transformed in view of the skewness) showed the difference between the treated and control group to be significant (p < 0.01). For details, see the ‘Supporting information’.

### Lung function, respiratory complaints and quality of life (FSII)

There was no effect of the intervention on the children’s lung function. No significant group difference was found for the reported episodes of wheezing, respiratory tract infections or for the health status of the children as measured with FSII at any time-point (p > 0.10).

### Intervention evaluation

Only 30% (n=9) of the parents completed the evaluation questionnaire. Everyone reported to be (very) happy with the intervention, the duration and time interval between the sessions, except 1 family who was not pleased with the contents of the intervention. Coaching was positively evaluated. The parents felt understood and that attention was given to their needs. One family reported that their coach did not discuss topics that were important for them. All except 1 family reported that they were (very) encouraged to stop PS in their children because of the intervention. Only 33% (n=3) of the responders made a’stop plan’ and 11% (n=1) of thought it was useful. Thirty tree percent (n=3) of the responders reported to find the feedback about the child’s urine cotinine concentration confronting and 78% (n=7) were convinced that they needed to stop PS in their children after the urine cotinine feedback.

## Discussion

The results of the PREPASE study demonstrate a beneficial effect of the intervention stop PS exposure at 6 and 9 months of study: 27% (unverified) or 23% (verified) of the parents in the intervention group stopped exposing their child(ren) at 6 months compared to 7% in the control group. Also, the degree of PS exposure in terms of amount of cigarettes smoked in the presence of the child decreased in both groups, but more in the intervention group than in the control group, suggesting that participation in the trial might have been a motivating factor for parents to stop or reduce PS in their children. No significant differences were seen for the secondary outcomes related to the child’s respiratory health. At 12 months, 6 months after stopping the intervention, no significant difference between intervention and control group was found any more on the primary outcome ‘exposure to PS’, which suggests that the effect of the intervention may decrease over time. Further, the large standard errors and wide confidence intervals for the effects imply that the present effect estimates are imprecise, due to the limited sample size.

Recently, a Cochrane review on the topic has been published, which concluded that the effectiveness of parental education and counselling programmes to reduce children’s tobacco smoke exposure was not clearly demonstrated^30^.

The PREPASE study suggests that a tailored intervention strategy with MI and feedback about the urine cotinine is an effective strategy to motivate parents to stop PS in their children. How can we explain this positive effect? In MI, the parents’ choice, personal responsibility for change, and enhancement of self-efficacy are emphasized. Counselling in the PREPASE study was focused on building motivation within parents, on empathy and giving confidence to parents. Possible barriers experienced by the parent(s) were discussed^21^.

Most parents in our study reported that they were happy with the intervention and the coaching. Also, many parents appreciated the feedback of the urine cotinine, although some parents experienced this part as intimidating.

Some other studies also found positive effects of MI and counselling^12,31–33^. In the study by Emmons *et al*., MI was effective to reduce household nicotine levels, but the long-term effects are not known as the study had a follow-up period of 6 months^12^. Recently, MI as part of an intervention program to protect children from PS resulted in an increased percentage of families smoking outdoors, decreased cigarette consumption and urine cotinine values of children^32^. Therefore, these studies showed that MI is an effective strategy to help parents stop PS exposure in their children, and should be included in such behavioural change programs. Moreover, a recent study with feedback about home air pollution and biomonitoring of hair nicotine of children was also effective in reducing PS in children^34^. However, providing feedback about urine cotinine as part of an intervention strategy was not effective in some studies^35,36^. Still, as with the PREPASE study, urine cotinine feedback was part of the intervention program and therefore it is not possible to interpret the effectiveness of the urine cotinine feedback.

The verified and unverified urine cotinine measures of PS in children showed a small discrepancy between the two measures, which was also observed in other studies^37,38^. In the PREPASE study, the differences between the two measures was mainly caused by the missing values for the urine cotinine. One family reported that the child’s urine cotinine was high (>10 μg/l) because the child had started to smoke. Yet, the conclusions of the two measures for the primary outcome were similar, a beneficial intervention effect after 6 and 9 months, but a smaller and insignificant influence after 12 months of study. A positive intervention effect immediately after the intervention period that disappeared at follow-up had been reported previously^18,38,39^. Apparently, for a long-lasting intervention effect, the duration of the program should be longer than 6 months.

### Limitations

Due to recruitment difficulties, the sample size was smaller than originally planned, and the results should be interpreted cautiously^21^. The social norms regarding PS in children have changed over the last years. Because of all the media attention and public awareness regarding PS, parents may have been more hesitant to participate in the study due to its confrontational topic. Also, PS in children is more prevalent in families of low social-economic status^40^. Reaching these families appeared to be very difficult. Furthermore, the PREPASE study had several drop-outs before the baseline measurement and additional missing values due to parents not being able to participate in every measurement, despite the incentives and efforts to retain the families in the study. By using mixed regression models for analyses, we were still able to include all participants with at least one measurement into the analyses, but not those 8 participants who dropped out between randomisation and baseline. Fortunately, a sensitivity analysis including those 8 participants by assuming they were still exposed to passive smoking after 3 months gave almost the same results as the analysis without those 8 participants. Further, the evidence for the intervention effect on exposure to passive smoking increased after adjusting for gender and after model reductions (see “Supporting information” for details). On the other hand, the width of the confidence interval for the intervention effect implies a rather imprecise effect estimate, which is due to the small sample size. Only 30% of the participants completed the questionnaire about the process evaluation and the results should be interpreted with caution as we are not informed about the opinion/experiences of the other families.

### Implications

The intervention effect of the PREPASE study might be maintained after 9 months by extending the intervention period beyond 6 months or adapt the duration to the specific need of a particular family. However, the major problem of the PREPASE study was the resistance of suitable families to participate. Future studies on this topic could focus more on how to overcome the defensive barrier of parents to participate in such studies, especially the hard-to-reach population of low social-economic status and/or heavy smokers. A Dutch study demonstrated that doctors infrequently discuss the topic of PS with parents^41^. In the PREPASE study, parents were more willing to participate when they were actively recruited via their physician. Therefore, physicians should have a more active role in stimulating parents to stop PS in their child(ren). Furthermore, PS exposure also exists in the form of third-hand smoke (THS), which is tobacco smoke residuals that remain long after the tobacco product has been extinguished, and parents should be further educated that the best way to eliminate all PS exposure in their children is by stopping active smoking^42,43^. Perhaps, an internet based program or a program offered at their primary care physician’s practice might be less intimidating for parents.

## Conclusion

The percentage of children exposed to PS at home was significantly lower in the intervention group than in the control group after 6 and 9 months of study. No significant group differences were seen after 3 and 12 months of study. These results suggest that MI combined with feedback of the child’s urine cotinine concentration may be an effective strategy to stop PS in children. A program longer than 6 months might be necessary for a longer lasting intervention effect. A limitation of the study was the sample size and the number of drop-outs and missing values.

## Electronic supplementary material

Supporting information
