# Supplementary material for: Motivational interviewing and urine cotinine feedback to stop passive smoke exposure in children predisposed to asthma: a randomised controlled trial
Source: Sci Rep. 2017 Nov 13;7:15473. doi: 10.1038/s41598-017-15158-2 (PMC5684321; doi:10.1038/s41598-017-15158-2)
Supplement: Supplementary file 1 — Supporting information [file 41598_2017_15158_MOESM1_ESM.pdf]

Motivational interviewing and urine cotinine feedback to stop passive smoke exposure in children predisposed to asthma: a randomised controlled trial

Supporting Information

Sasha G Hutchinson[1], MD, PhD: sasha.hutchinson@maastrichtuniversity.nl;

Gerard van Breukelen[2], Professor: gerard.vbreukelen@maastrichtuniversity.nl;

Constant P van Schayck[3], Professor: onno.vanschayck@maastrichtunversity.nl;

Brigitte Essers[4], PhD: brigitte.essers@mumc.nl;

S. Katharine Hammond[5], Professor: hammondk@berkeley.edu;

Jean WM Muris[3], Professor, MD: jean.muris@maastrichtuniversity.nl;

Frans JM Feron[6], Professor, MD: f.feron@maastrichtuniversity.nl;

Edward Dompeling[1], Professor, MD: edward.dompeling@mumc.nl.

Affiliations:

1. Department of Paediatric Pulmonology, Maastricht University Medical Center (MUMC+) / CAPHRI School for Public Health and Primary Care, P.O. Box 616, 6200 MD Maastricht, the Netherlands.
2. Department of Methodology and Statistics, MUMC+ / CAPHRI, P.O. Box 616, 6200 MD Maastricht, the Netherlands.
3. Department of Family Medicine, MUMC+ / CAPHRI, P.O. Box 616, 6200 MD Maastricht, the Netherlands.
4. Department of Clinical Epidemiology and Medical Technology Assessment, MUMC+.
5. School for Public Health, University of California, 50 University Hall 7360, Berkeley, CA 94720-7360
6. Department of Social Medicine, MUMC+ / CAPHRI, P.O. Box 616, 6200 MD Maastricht, the Netherlands.

Address correspondence to: Sasha Hutchinson, Department of Paediatric Pulmonology, Maastricht University Medical Center, P.O. Box 616, 6200 MD, Maastricht, Netherlands. (sasha.hutchinson@maastrichtuniversity.nl).

Methods

Intervention effect

The intervention effect on the primary outcomes (urine cotinine verified and not verified parental report of PS exposure (yes (1) and no (0) in their children at home) were analysed with: a) Chi-square tests of the group difference per time point, in particular after six months, as preliminary analysis, and b) mixed logistic regression analysis (using Generalized Linear Mixed-effects Models (GLMM) in SPSS) of all repeated measures to allow inclusion of all participants with at least one measurement of exposure and to take the correlations between the repeated measurements into account, as the main analysis, using a two-tailed $\alpha = 0.05$. Missing values were not imputed, except in some sensitivity analyses that served to check the robustness of the primary analyses.

Statistical Analysis

For both primary outcome measures, the fixed part of the mixed logistic model contained intercept, group (control (0) versus intervention (1)), time (3, 6, 9, and 12 months using dummy indicator coding, and interaction terms of group by each time indicator. The random part of the model consisted of an unstructured covariance matrix for the repeated measures (which is the most general structure and fitted better than alternative, more simple, structures). Additionally, the possible effects of the child's age and gender, and parental social economic status on the risk of PS exposure were analysed. For each of the two definitions for the primary outcome as mentioned above, a sensitivity analysis was performed by replacing a missing value for PS exposure after 3 months due to dropout with PS exposure=yes (as at baseline) to allow inclusion of all randomized children, without replacing missing values after 6,9 and 12 months. Remember that the baseline measurement of the primary outcome exposure to passive smoking could not be included into the mixed logistic regression because all patients were exposed at baseline by definition, due to the inclusion criteria.

The secondary outcomes were analysed with mixed linear regression (continuous variables) or mixed logistic regression (binary outcomes), following the same modelling procedures as for the primary outcomes, except that the baseline recording was now

included. Further, each secondary outcome was tested with a two-tailed α = 0.01 instead of 0.05 to reduce the risk of false positives due to multiple outcome testing. The data for the number of cigarettes/day indoors in the presence of the child per day, and for the total number of cigarettes/day were transformed by taking their square root in view of severe non-normality of the residuals.

Results

Intervention effect (primary outcome)

The results of the mixed logistic regression analysis of the primary outcome Exposure to passive smoking (cotinine verified and not-verfied) are shown in Table 3. The results of the sensitivity analysis were very close to these results. In particular, after 6 months the group difference on validated exposure was B = -1.67 (SE = 0.92, p = 0.07) on the log odds scale, implying an odds ratio of 0.19 for treated versus controls or 5.31 for controls versus treated, in the sensitivity analysis. For the not-verified exposure it was B = -1.74 (SE = 0.86, p = 0.04), giving an odds ratio of 0.18 resp. 5.70. These results are close to those in Table 3.

Further, to improve stability of the effect estimates and standard errors, the mixed model reported in Table 3 was compared with model simplifications in the random part (unstructured versus compound symmetry) and fixed part (constant group difference, linear and/or quadratic divergence) by means of the Akaike and Bayesian information criteria. This showed that a simple GLMM with a compound symmetric covariance structure and a constant group difference from month 3 till month 12 on the log odds scale fitted better than any other model for the cotinine verified and for not-verified exposure. This model gave the following results, which apply to each time point except baseline provided the simplified model is valid: for the cotinine verified group difference was estimated to be B = -1.96 (SE = 0.62, p = 0.002) on the log odds scale, implying an odds ratio of 0.14 (95% CI from 0.04 to 0.49) for exposure among treated versus controls, or an odds ratio of 7.10 (95% CI from 2.05 to 24.53) for exposure among controls versus treated. For the not-verified exposure the effect was B = -1.49 (SE = 0.54, p = 0.008) on the log odds scale, implying an odds ratio of 0.23 (95% CI from 0.08 to 0.66) for exposure

among treated versus controls, or an odds ratio of 4.44 (95% CI from 1.51 to 13.07) for exposure among controls versus treated. These numbers suggest a substantive beneficial treatment effect, but the width of the confidence intervals shows the effect estimates to be very imprecise, due to the small sample size. Compared with the standard errors in Table 3 based on the initial model, the present results are more precise, however.

Finally, additional analyses explored the possible effect of the child's age, gender and the parents' socio-economic status on the risk of PS exposure. Only a significant effect of gender was found ($p<0.01$, higher exposure of girls). Further tests of gender by group and gender by time interactions did not show any significant interactions. Moreover, gender was not a confounder (see Table 1). Including gender as a main effect into the initial GLMM as reported in Table 3 gave the following group difference after 6 months: For validated exposure B = -2.24 (SE = 1.05, p = 0.035) and an odds ratio of 0.11 for treated versus controls resp. 9.39 for controls versus treated . For the not-verified exposure B = -2.16 (SE = 0.85, p = 0.013) and an odds ratio of 0.12 resp. 8.67.

Number of cigarettes smoked in the presence of the child

Mixed linear regression analysis was performed on the square root transformed number of cigarettes smoked per day indoors in the child's presence, using the same modelling procedure as for the primary outcomes. The resulting predicted outcome mean is plotted in Figure 2 panel B, and gives the best estimate of the time course of this outcome per group as it adjusts for bias due to drop-out of the missing at random (MAR) type, whereas plots of the observed data require the stronger missing completely at random (MCAR) assumption.[22] Comparisons of the initial mixed model with simplified models using likelihood ratio testing, showed that the group difference in terms of the square root number of cigarettes was constant at 3, 6, 9, and 12 months of study (estimated difference: β=-0.90, Standard Error (SE)=0.30, 95% Confidence Interval (CI): -1.51 - -0.29, $p<0.01$). The number of missing values for this outcome was 31% however, and so the present results must be interpreted with caution as these are based on the MAR missingness assumption.

Parental active smoking

No group differences were observed for the number of primary caregivers reporting to have no intention to quit active smoking. After 6 months 10% (n=3) in the intervention group and 14% (n=4) in the control group reported to have no intention to stop smoking, and this remained the same after 12 months of study. One family (3%) in the intervention group reported to have quit smoking after the second counselling session (between baseline and 3 months of study) and reported no relapse. The urine cotinine concentration in their child was <3 μg/l at time-point 6-12 months. Two families in the control group reported to have stopped active smoking, one at 9 and the other at 12 months of follow-up. However, this was not confirmed by the child's urine cotinine. Similar to the number of cigarettes smoked indoors in the child's presence, the total number of cigarettes smoked per day was strongly skewed and so we plotted its median instead of its mean against time (Figure 2 panel C) and analysed its square root with mixed linear regression using the same modelling procedure as before, and plotted the predicted values from the mixed regression (Figure 2 panel D). Likelihood ratio comparisons between different mixed models showed the group difference with respect to this outcome to increase linearly over time ($\beta = -0.21$ = the increase in group difference per quarter, SE = 0.09, 95% CI: -0.40 - -0.2, $0.01<p<0.05$, which just fails to be significant by the α of 0.01 planned for secondary outcomes), see Figure 2 panel D. The number of missing values were 28%, and so the present results must again be treated with caution as they depend on the validity of the MAR missingness assumption.
